# Supplementary material for: Enhancing shift current response via virtual multiband transitions
Source: Commun Phys. 2024 Jul 20;7(1):250. doi: 10.1038/s42005-024-01729-z (PMC11271335; doi:10.1038/s42005-024-01729-z)
Supplement: Supplementary file 2 — Supplementary Materials [file 42005_2024_1729_MOESM2_ESM.pdf]

Supplementary material for "Enhancing Shift Current Response via Virtual Multiband Transitions"

Sihan Chen,[1,2] Swati Chaudhary,[3,4,5] Gil Refael,[2,6] and Cyprian Lewandowski[7,8]

[1]Kadanoff Center for Theoretical Physics, University of Chicago, Chicago, Illinois 60637, USA
[2]Department of Physics, California Institute of Technology, Pasadena CA 91125, USA
[3]Department of Physics, The University of Texas at Austin, Austin, Texas 78712, USA
[4]Department of Physics, Northeastern University, Boston, Massachusetts 02115, USA
[5]Department of Physics, Massachusetts Institute of Technology, Cambridge, Massachusetts 02139, USA
[6]Institute for Quantum Information and Matter, California Institute of Technology, Pasadena CA 91125, USA
[7]National High Magnetic Field Laboratory, Tallahassee, Florida, 32310, USA
[8]Department of Physics, Florida State University, Tallahassee, Florida 32306, USA

Supplementary Note 1: Deriving shift current integrand expression

For completeness, we include the derivation of the shift current integrand used in the main text. In general, there are two frameworks people use to compute shift current under independent electron and dipole fields approximation. In the length gauge, one uses position operator $\mathbf{r}$ and modifies the Hamiltonian $H \to H - e\mathbf{E}\cdot\mathbf{r}$. In the velocity gauge, one modifies the Hamiltonian via minimal coupling $H \to H(\mathbf{k} - e\mathbf{A})$. [1] The two frameworks are related to each other via a generalized sum rule (e.g. See Ref. [2] and discussion therein.). The expression of shift current, derived using length gauge in [3], can be written in terms of dipole matrix element r^α_{mn} and its generalized derivative $r^\alpha_{mn;\beta}$ is given by

$$\sigma^{\alpha\alpha}_{\beta}(0;\omega,-\omega) = \frac{-i\pi e^3}{\hbar^2}\int_{\mathrm{BZ}}\sum_{mn} f_{mn} r^\alpha_{mn;\beta} r^\alpha_{mn}\delta(\omega_{mn}-\omega), \tag{S.1}$$

where

$$r^\alpha_{mn} = A^\alpha_{mn} \qquad m \neq n \text{ and } 0 \text{ otherwise} \tag{S.2}$$

$$r^\alpha_{mn;\beta} = \frac{\partial r^\alpha_{mn}}{\partial k^\beta} - i(A^\beta_{mm} - A^\beta_{nn})r^\alpha_{mn}. \tag{S.3}$$

To connect the length gauge expression of Eq. S.1 to the velocity gauge expression used in the main text, we use generalized sum rule for generalized derivative provided in [2]

$$r^\alpha_{mn;\beta} = -\frac{1}{i\varepsilon_{mn}}\left[\frac{h^\alpha_{mn}\Delta^\beta_{mn} + h^\beta_{mn}\Delta^\alpha_{mn}}{\varepsilon_{mn}} - w^{\alpha\beta}_{mn} + \sum_{l\neq m,n}\left(\frac{h^\alpha_{ml}h^\beta_{ln}}{\varepsilon_{ln}} - \frac{h^\beta_{ml}h^\alpha_{ln}}{\varepsilon_{ml}}\right)\right], \tag{S.4}$$

where $h^\alpha_{mn} = \langle m|\partial_{k_\alpha}H|n\rangle$, $w^{\alpha\beta}_{mn} = \langle m|\partial_{k_\alpha}\partial_{k_\beta}H|n\rangle$. Using $r^\alpha_{nm} = -ih^\alpha_{nm}/\varepsilon_{nm}$, the shift current integrand as defined in the main text is then

$$R^{\alpha\alpha\beta}_{mn} = \mathrm{Im}\left[r^\alpha_{mn;\beta}r^\alpha_{nm}\right] \tag{S.5}$$

$$= \frac{1}{\varepsilon^2_{mn}}\mathrm{Im}\left[-\frac{h^\alpha_{nm}h^\alpha_{mn}\Delta^\beta_{mn} + h^\alpha_{nm}h^\beta_{mn}\Delta^\alpha_{mn}}{\varepsilon_{mn}} + w^{\alpha\beta}_{mn}h^\alpha_{nm}\right] \tag{S.6}$$

$$+ \frac{1}{\varepsilon^2_{mn}}\mathrm{Im}\left[\sum_{l\neq mn}\left(\frac{h^\alpha_{nm}h^\beta_{ml}h^\alpha_{ln}}{\varepsilon_{ml}} - \frac{h^\alpha_{nm}h^\alpha_{ml}h^\beta_{ln}}{\varepsilon_{ln}}\right)\right]. \tag{S.7}$$

Noting that $h^\alpha_{nm}h^\alpha_{mn} = |h^\alpha_{mn}|^2$ and $\mathrm{Im}[h^\alpha_{mn}h^\beta_{nm}] = -\mathrm{Im}[h^\alpha_{nm}h^\beta_{mn}]$, we recover the expression in the main text:

$$R^{\alpha\alpha\beta}_{mn} = \frac{1}{\varepsilon^2_{mn}}\mathrm{Im}\left[\frac{h^\alpha_{mn}h^\beta_{nm}\Delta^\alpha_{mn}}{\varepsilon_{mn}} + w^{\alpha\beta}_{mn}h^\alpha_{nm}\right] + \frac{1}{\varepsilon^2_{mn}}\mathrm{Im}\left[\sum_{l\neq mn}\left(\frac{h^\alpha_{nm}h^\beta_{ml}h^\alpha_{ln}}{\varepsilon_{ml}} - \frac{h^\alpha_{nm}h^\alpha_{ml}h^\beta_{ln}}{\varepsilon_{ln}}\right)\right]. \tag{S.8}$$

Supplementary Note 2: Fubini-Study metric

The quantum geometric tensor provide a tool for us to describe the geometry of eigenstate. In the context of bandstructure, the quantum geometric tensor[4, 5] for a single band m is

$$G^m_{\mu\nu}(k) = \langle \partial_\mu m(k)| (1 - |m(k)\rangle \langle m(k)|) |\partial_\nu m(k)\rangle \tag{S.9}$$

$$= \sum_{n\neq m} \langle \partial_\mu m(k)|n(k)\rangle \langle n(k)|\partial_\nu m(k)\rangle \tag{S.10}$$

$$= \sum_{n\neq m} \frac{\langle m|\partial_\mu H|n\rangle \langle n|\partial_\nu H|m\rangle}{(E_m - E_n)^2} \tag{S.11}$$

Under this definition, we find

$$G^m_{\mu\nu}(k) = \mathrm{Re}[G^m_{\mu\nu}(k)] + i\mathrm{Im}[G^m_{\mu\nu}(k)] = g^m_{\mu\nu}(k) - \frac{i}{2}F^m_{\mu\nu}, \tag{S.12}$$

with the real part being the symmetric Fubini-Study (FS) metric and the imaginary part being the antisymmetric Berry curvature[4, 5], given by

$$g^m_{\mu\nu} = \frac{1}{2}\sum_{n\neq m} \frac{\langle m|\partial_\mu H|n\rangle \langle n|\partial_\nu H|m\rangle + \langle m|\partial_\nu H|n\rangle \langle n|\partial_\mu H|m\rangle}{(E_m - E_n)^2}, \tag{S.13}$$

$$F^m_{\mu\nu} = i\sum_{n\neq m} \frac{\langle m|\partial_\mu H|n\rangle \langle n|\partial_\nu H|m\rangle - \langle m|\partial_\nu H|n\rangle \langle n|\partial_\mu H|m\rangle}{(E_m - E_n)^2} \tag{S.14}$$

As pointed out in [6], the FS metric characterizes the spread of Wannier wavefunction. Following the definition of Ref.[6], we define the localization of Wannier wavefunciton as

$$F = \sum_n \left[\langle 0n|\hat{\mathbf{r}}^2|0n\rangle - |\langle 0n|\hat{\mathbf{r}}|0n\rangle|^2 \right], \tag{S.15}$$

where $\hat{\mathbf{r}}$ is the position operator and $|\mathbf{R}n\rangle$ is the Wannier state given by $|\mathbf{R}n\rangle = \frac{1}{\sqrt{N}}\sum_{\mathbf{k}} e^{-i\mathbf{k}\cdot\mathbf{R}} |\psi_{n\mathbf{k}}\rangle$. Then, FS metric provides a lower bound on F given by

$$F \geq \frac{\Omega_c}{(2\pi)^2}\int d^2k\, \mathrm{tr}\, g(k), \tag{S.16}$$

with Ω_c being the area of the unit cell and $g_{\mu\nu}(k) = \sum_m g^m_{\mu\nu}(k)$.

The FS metric defined above refers to a single band m and sums over contribution from all intermediate band n. Without summing over n, we define the so-called band-resolved quantum metric[7] as

$$Q^{mn}_{\mu\nu}(k) = \langle \partial_\mu m(k)|n(k)\rangle \langle n(k)|\partial_\nu m(k)\rangle \tag{S.17}$$

$$= \frac{\langle m|\partial_\mu H|n\rangle \langle n|\partial_\nu H|m\rangle}{(E_m - E_n)^2}. \tag{S.18}$$

We can consequently define band-resolved FS metric and Berry curvature as

$$g^{mn}_{\mu\nu}(k) = \mathrm{Re}[Q^{mn}_{\mu\nu}(k)], \qquad F^{mn}_{\mu\nu} = -2\mathrm{Im}[G^{mn}_{\mu\nu}(k)]. \tag{S.19}$$

In terms of projector notations, we can write

$$G^{mn}_{\mu\nu} = \mathrm{Tr}[P_n\partial_\mu P_m\partial_\nu P_m] = \mathrm{Tr}[P_m\partial_\mu P_{nm}\partial_\nu P_{mn}], \tag{S.20}$$

where $P_m = |m\rangle\langle n|$, and $P_{mn} = |m\rangle\langle n|$.

In 1D, there is no interband Berry curvature since G^{mn}_{xx} is real. Furthermore, the transition probability amplitude can be identified with interband FS metric in the direction of transition:

$$|A^\alpha_{mn}|^2 = -\langle m(k)|\partial_\alpha n(k)\rangle \langle n(k)|\partial_\alpha m(k)\rangle = g^{\alpha\alpha}_{mn}. \tag{S.21}$$

This allows us to write the shift current expression of Eq. 2 as

$$\sigma^{\beta}_{\alpha\alpha}(\omega) = \frac{\pi e^3}{\hbar^2} \sum_{m,n} \int d^2\mathbf{k} f_{mn} S^{\beta\alpha}_{mn} g^{\alpha\alpha}_{mn} \delta(\omega - \varepsilon_{mn}), \tag{S.22}$$

where we establish a direct connection between shift current response and FS metric. Since $\sum_{mn\alpha} \int d\mathbf{k} g^{\alpha\alpha}_{mn}$ gives a lower bound for the spread of Wannier wavefunction, this confirms many of the experimental evidence[8, 9] that materials with delocalized wavefunction tend to exhibit large shift current response. In addition, viewing $g^{\alpha\alpha}_{mn}$ as the α component of the metric, we can view shift current conductivity tensor as the sum of shift vector at each k-state over the BZ weighted by the interband metric along the direction of transition.

Supplementary Note 3: Resonance of the Quantum Geometric Tensor in Parameter Space

Near the parameter region where the band gap vanishes at the band edge, we expect g^{mn}_{xx} to diverge. We describe the rate of divergence by a Lorentzian function

$$L(x) = \frac{2A}{\pi} \frac{\Gamma}{4(x - x_0)^2 + \Gamma^2} + c, \tag{S.23}$$

with constant A, x_0, Γ, c. For the 2RM model considered in the main text, we fix parameter $(t, \delta_1, \Delta_1, \Delta_2, \varepsilon)$ and vary δ_2 to optimize for shift current enhancement via virtual transitions, and we note that the divergence of FS metric at the band edge as $\delta_2 \to \delta_{2,0}$ is well-characterized by a Lorentzian function, where $\delta_{2,0}$ is where the gap is minimized.

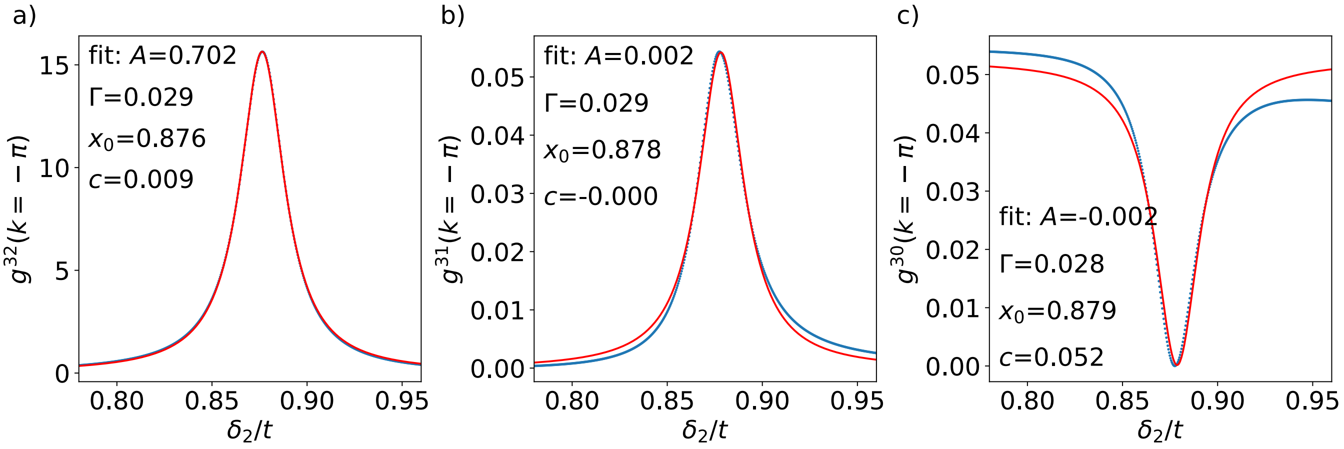

Supplementary Figure 1. The FS metric $g^{mn}_{xx}(k)$ at the band edge $ka = -\pi$ for $m = 3$, $n = 2, 1, 0$, and the fit using a Lorentzian function, with fitting curve in red and exact calculation in blue dots. Here we used the same parameters as in the main text, namely $(t, \delta_1, \Delta_1, \Delta_2, \varepsilon) = (1, 0.8, 0.7, 0.6, 0.1)$.

We plot the component of g^{mn} at the band edge for the upper conduction band $m = 3$ and $n = 0, 1, 2$ in Fig. Supplementary Figure 1a,b,c. For $g^{32}(k)$, the Lorentzian function well-characterizes the divergence at the band edge as $\delta_{2,0}$ is approached. For $g^{31}(k)$ and $g^{30}(k)$, the tails show deviations from Lorentzian behavior, but near the resonance pole, they can be described by the Lorentzian function. It is this divergence structure that gives rise to enhanced $g_3(k) = \sum_{n\neq 3} g^{3n}(k)$ near $\delta_{2,0}$.

This evokes the resonance phenomena but in parameter space. As we vary the hopping parameters, there exists a critical hopping strength such that the FS metric diverges at the band edge. This enhances the sum of FS over all k-states, leading to Wannier wavefunction being resonantly more delocalized. Consequently, we observe an enhancement in shift current response via virtual transitions.

Now, we demonstrate this connection more precisely by explicitly putting g^{mn} into the form of a Lorentzian. The

exact energy eigenvalues of 2RM model is given by

$$a = \Delta_1^2 + \Delta_2^2 + \delta_1^2 \sin^2\left(\frac{k}{2}\right) + \delta_2^2 \sin^2\left(\frac{k}{2}\right) + 2t^2 \cos^2\left(\frac{k}{2}\right), \tag{S.24}$$

$$b = 4t^2 \cos^2\left(\frac{k}{2}\right) + (\Delta_1 - \Delta_2)^2 + (\delta_1 - \delta_2)^2 \sin^2\left(\frac{k}{2}\right), \tag{S.25}$$

$$c = (\Delta_1 - \Delta_2)(\Delta_1 + \Delta_2) + (\delta_1 - \delta_2)(\delta_1 + \delta_2) \sin^2\left(\frac{k}{2}\right), \tag{S.26}$$

$$E = \pm \frac{\sqrt{2}}{2} \sqrt{a + 2\varepsilon^2 \pm \sqrt{4\varepsilon^2 b + c^2}}. \tag{S.27}$$

Recall that the FS metric tensor defined in Appendix 2 in 1D is given by

$$g^{mn}(k) = \frac{\langle m|\partial_k H|n\rangle \langle n|\partial_k H|m\rangle}{(E_m - E_n)^2}. \tag{S.28}$$

Using $m = 3$, $n = 2$ as an example and focus on the band edge at $k = \pm\pi$, we put the above FS in the form of Lorentzian explicitly. Noting that there are no poles in the numerator, so we focus on the denominator term, which gives

$$(E_3 - E_2)^2 = a + 2\varepsilon^2 - \sqrt{(a + 2\varepsilon^2)^2 - 4\varepsilon^2 b + c^2} \approx \frac{1}{2} \frac{4\varepsilon^2 b + c^2}{(a + 2\varepsilon^2)}, \tag{S.29}$$

where we note that $4\varepsilon^2 b + c^2 \ll (a + 2\varepsilon^2)^2$ near the pole. The pole occurs in $4\varepsilon^2 b + c^2$. We expand δ_2 around the maximum of $g^{32}(k)$, denoted by $\delta_{2,0}$, to second order, and we find

$$4\varepsilon^2 b + c^2 = \tilde{a}\left(\delta_2 - \delta_{2,0} + \frac{\tilde{b}}{2\tilde{a}}\right)^2 - \frac{\tilde{b}^2}{4\tilde{a}} + \tilde{c} + \mathcal{O}((\delta_2 - \delta_{2,0})^2), \tag{S.30}$$

with

$$\tilde{a} = -2(\delta_1^2 + \Delta_1^2 - \Delta_2^2 - 3\delta_{2,0}^2 - 2\varepsilon^2) \tag{S.31}$$

$$\tilde{b} = -4(\delta_{2,0}(\delta_1^2 + \Delta_1^2 - \Delta_2^2 - \delta_{2,0}^2) + 2(\delta_1 - \delta_{2,0})\varepsilon^2) \tag{S.32}$$

$$\tilde{c} = (\delta_1^2 + \Delta_1^2 - \Delta_2^2 - \delta_{2,0}^2)^2 + 4((\Delta_1 - \Delta_2)^2 + (\delta_1 - \delta_{2,0})^2)\varepsilon^2. \tag{S.33}$$

By the assumption that $\delta_{2,0}$ is the pole, we require $\frac{\tilde{b}}{2\tilde{a}} = 0$. Alternatively, the value of $\delta_{2,0}$ is obtained by solving for $\frac{\tilde{b}}{2\tilde{a}} = 0$. The FS metric g^{32} at $k = \pm\pi$ as a function of hopping strength δ_2 can be written in the form of a Lorenzian

$$g^{32}(\delta_2) = \frac{2A}{\pi} \frac{\Gamma}{4(\delta_2 - \delta_{2,0})^2 + \Gamma^2}, \tag{S.34}$$

with the width and amplitude given by

$$\Gamma = 2\sqrt{\frac{\tilde{c}}{\tilde{a}}}, \qquad A = \frac{4\pi(a + 2\varepsilon^2)|\langle 3|\partial_k H|2\rangle|^2}{\tilde{a}\Gamma}. \tag{S.35}$$

A similar manipulation follows for the case of $n = 1$ and $n = 0$. The Γ terms acts like a damping term that restricts the divergences of FS at the band edge. This Γ damping term usually occurs in systems with many parameters, and may be absent in models with fewer parameters. Consider the Su–Schrieffer–Heeger (SSH) model[10], with Hamiltonian given by

$$H(k) = h_x \sigma_x + h_y \sigma_y, \qquad h_x = v + w\cos(k), \qquad h_y = w\sin(k), \tag{S.36}$$

where v is the intralayer hopping amplitude and w is the interlayer hopping amplitude. Fixing interlayer hopping w and vary intralayer hopping v, the FS metric at $k = \pm\pi$ is given by

$$g(v) = \frac{w^2}{4(v - w)^2}, \tag{S.37}$$

where it diverges without damping at $v = w$, the point when energy gap closes. Adding a mass term Δ to break the chiral symmetry, we obtain a single-layer Rice-Mele Model, and the damping in FS metric becomes 4Δ.

Supplementary Note 4: Analytical Results of 2RM model

In the main text, we established that it is the enhancement in virtual transitions near the critical parameter region that leads to enhanced shift current response. Inspecting Eq. 3 shows us the two factors that control of the magnitude of the system are energy gap ε_{mn} and matrix element $h_{mn} = \langle m|\partial_k H|n\rangle$. In particular, we expect ε_{32} to dominate in the denominator when the gap is small, and we will show that it is divergence in $\mathrm{Im}\{h_{20}\}$ that dominates in the numerator.

The Hamiltonian of 2RM model takes the form $H = H_0 + V$, with

$$H_0 = \begin{pmatrix} d_z^{(1)} & d_x^{(1)} - id_y^{(1)} & 0 & 0 \\ d_x^{(1)} + id_y^{(1)} & -d_z^{(1)} & 0 & 0 \\ 0 & 0 & d_z^{(2)} & d_x^{(2)} - id_y^{(2)} \\ 0 & 0 & d_x^{(2)} + id_y^{(2)} & -d_z^{(2)} \end{pmatrix}, \quad V = \begin{pmatrix} 0 & 0 & 0 & \varepsilon \\ 0 & 0 & \varepsilon & 0 \\ 0 & \varepsilon & 0 & 0 \\ \varepsilon & 0 & 0 & 0 \end{pmatrix}, \tag{S.38}$$

where H is in the layer basis $\{|1A\rangle, |1B\rangle, |2A\rangle, |2B\rangle\}$, and superscript (1/2) denotes the first/second RM model. Exactly solving for the eigenstate and computing matrix element will result in complicated expressions that shed little intuition. Instead, we will use perturbation theory and expand near the band edge. A direct application of non-degenerate perturbation theory leads to inaccurate results near the band edge; hence, we first perform a basis transformation to the unperturbed eigenstate $\{|\psi_{1,A}\rangle, |\psi_{2,A}\rangle, |\psi_{1,B}\rangle, |\psi_{2,B}\rangle\}$. The Hamiltonian $\tilde{H} = \tilde{H}_0 + \tilde{V}$ becomes

$$\tilde{H}_0 = \begin{pmatrix} -\varepsilon_1(k) & f_A(k) & 0 & 0 \\ f_A^*(k) & -\varepsilon_2(k) & 0 & 0 \\ 0 & 0 & \varepsilon_1(k) & f_B(k) \\ 0 & 0 & f_B^*(k) & \varepsilon_2(k) \end{pmatrix}, \quad \tilde{V} = \begin{pmatrix} 0 & 0 & 0 & g_1(k) \\ 0 & 0 & g_2(k) & 0 \\ 0 & g_2^*(k) & 0 & 0 \\ g_1^*(k) & 0 & 0 & 0 \end{pmatrix}, \tag{S.39}$$

where $\varepsilon_{1/2}$ is the eigenvalue of the first/second unperturbed RM model, and

$$f_A(k) = \langle\psi_{1,A}|V|\psi_{2A}\rangle = \frac{\varepsilon}{2\sqrt{\varepsilon_1\varepsilon_2}}\left(-e^{i\varphi_k^{(2)}}\sqrt{\varepsilon_1 - d_z^{(1)}}\sqrt{\varepsilon_2 + d_z^{(2)}} - e^{-i\varphi_k^{(1)}}\sqrt{\varepsilon_1 + d_z^{(1)}}\sqrt{\varepsilon_2 - d_z^{(2)}}\right), \tag{S.40}$$

$$f_B(k) = \langle\psi_{1,B}|V|\psi_{2B}\rangle = \frac{\varepsilon}{2\sqrt{\varepsilon_1\varepsilon_2}}\left(e^{-i\varphi_k^{(1)}}\sqrt{\varepsilon_1 - d_z^{(1)}}\sqrt{\varepsilon_2 + d_z^{(2)}} + e^{i\varphi_k^{(2)}}\sqrt{\varepsilon_1 + d_z^{(1)}}\sqrt{\varepsilon_2 - d_z^{(2)}}\right), \tag{S.41}$$

$$g_1(k) = \langle\psi_{1,A}|V|\psi_{2B}\rangle = \frac{\varepsilon}{2\sqrt{\varepsilon_1\varepsilon_2}}\left(-e^{i\varphi_k^{(2)}}\sqrt{\varepsilon_1 - d_z^{(1)}}\sqrt{\varepsilon_2 - d_z^{(2)}} + e^{-i\varphi_k^{(1)}}\sqrt{\varepsilon_1 + d_z^{(1)}}\sqrt{\varepsilon_2 + d_z^{(2)}}\right), \tag{S.42}$$

$$g_2(k) = \langle\psi_{2,A}|V|\psi_{1B}\rangle = \frac{\varepsilon}{2\sqrt{\varepsilon_1\varepsilon_2}}\left(-e^{i\varphi_k^{(1)}}\sqrt{\varepsilon_1 - d_z^{(1)}}\sqrt{\varepsilon_2 - d_z^{(2)}} + e^{-i\varphi_k^{(2)}}\sqrt{\varepsilon_1 + d_z^{(1)}}\sqrt{\varepsilon_2 + d_z^{(2)}}\right), \tag{S.43}$$

where $\varphi_k^{(1/2)} = \arctan\left(d_y^{(1/2)}/d_x^{(1/2)}\right)$. Now, we can apply the non-degenerate perturbation theory safely. In particular, the first order correction in state is given by

$$|n\rangle = |n_0\rangle + \sum_{k\neq n}\frac{\langle k_0|V|n_0\rangle}{E_n - E_k}|k_0\rangle, \tag{S.44}$$

with nonzero matrix element only in

$$\langle 1_0|\tilde{V}|3_0\rangle = \frac{1}{2\sqrt{E_d^{(A)}E_d^{(B)}}}\Big(- g_1(k)e^{i\varphi_k^{(B)}}\sqrt{E_d^{(A)}+d_3^{(A)}}\sqrt{E_d^{(B)}+d_3^{(B)}} - g_2(k)e^{-i\varphi_k^{(A)}}\sqrt{E_d^{(A)}-d_3^{(A)}}\sqrt{E_d^{(B)}-d_3^{(B)}}\Big), \tag{S.45}$$

$$\langle 2_0|\tilde{V}|3_0\rangle = \frac{1}{2\sqrt{E_d^{(A)}E_d^{(B)}}}\Big(g_1(k)e^{i\varphi_k^{(B)}}\sqrt{E_d^{(A)}-d_3^{(A)}}\sqrt{E_d^{(B)}+d_3^{(B)}} - g_2(k)e^{-i\varphi_k^{(A)}}\sqrt{E_d^{(A)}+d_3^{(A)}}\sqrt{E_d^{(B)}-d_3^{(B)}}\Big), \tag{S.46}$$

$$\langle 1_0|\tilde{V}|4_0\rangle = \frac{1}{2\sqrt{E_d^{(A)}E_d^{(B)}}}\Big(- g_1(k)e^{i\varphi_k^{(B)}}\sqrt{E_d^{(A)}+d_3^{(A)}}\sqrt{E_d^{(B)}-d_3^{(B)}} + g_2(k)e^{-i\varphi_k^{(A)}}\sqrt{E_d^{(A)}-d_3^{(A)}}\sqrt{E_d^{(B)}+d_3^{(B)}}\Big), \tag{S.47}$$

$$\langle 2_0|\tilde{V}|4_0\rangle = \frac{1}{2\sqrt{E_d^{(A)}E_d^{(B)}}}\Big(g_1(k)e^{i\varphi_k^{(B)}}\sqrt{E_d^{(A)}-d_3^{(A)}}\sqrt{E_d^{(B)}-d_3^{(B)}} + g_2(k)e^{-i\varphi_k^{(A)}}\sqrt{E_d^{(A)}+d_3^{(A)}}\sqrt{E_d^{(B)}+d_3^{(B)}}\Big), \tag{S.48}$$

where

$$d_0 = \frac{\varepsilon_1(k)+\varepsilon_2(k)}{2}, \quad d_1^{(A/B)} = \mathrm{Re}\{f_{A/B}(k)\}, \quad d_2^{(A/B)} = \mathrm{Im}\{f_{A/B}(k)\}, \quad d_3 = \frac{\varepsilon_1(k)-\varepsilon_2(k)}{2}, \tag{S.49}$$

$$E_d^{(A/B)} = \sqrt{\left(d_1^{(A/B)}\right)^2 + \left(d_2^{(A/B)}\right)^2 + d_3^2}, \quad \varphi_k^{(A/B)} = \arctan\Big(d_2^{(A/B)}/d_1^{(A/B)}\Big) \tag{S.50}$$

In particular, there is no matrix element connecting the same block of $\tilde{H}_0$ where the difference in energy is small, thus avoiding the divergence in a small energy gap near gap closing. Comparing the perturbation theory result with exact diagonalization indeed shows the validity of this approach. The $\tilde{H}_0$ now becomes an effective 2-band model, which shift current response is shown in Fig. 2d. Comparing with tight-binding 2-band model, this effective 2-band model can achieve a large joint density of states and dispersive band structure simultaneously.

Now, we have the ingredients necessary to compute matrix element h_{mn}. For our consideration, take $m = 3, n = 0$. At the band edge, comparing the magnitude of direct and virtual transition contribution to shift current amounts to comparing $\mathrm{Re}\{w_{30}\}$ and $\mathrm{Re}\{h_{32}h_{20}\} = \mathrm{Im}\{h_{32}\}\mathrm{Im}\{h_{20}\}$ (in the integrand we are taking the Im of the expression, and $\mathrm{Re}\{h_{03}\} = 0$ at band edge). We find

$$\mathrm{Re}\{w_{30}\} = \langle 3_0|\partial_k^2\tilde{H}|0_0\rangle + \frac{\langle 3_0|\tilde{V}|0_0\rangle}{E_{30}}\Big[\langle 0_0|\partial_k^2\tilde{H}|0_0\rangle - \langle 3_0|\partial_k^2\tilde{H}|3_0\rangle\Big], \tag{S.51}$$

$$\mathrm{Im}\{h_{32}\} = \frac{\langle 1_0|\partial_k\tilde{H}|2_0\rangle\,\langle 3_0|\tilde{V}|1_0\rangle}{E_{31}} + \frac{\langle 0_0|\partial_k\tilde{H}|2_0\rangle\,\langle 3_0|\tilde{V}|0_0\rangle}{E_{30}} + \frac{\langle 3_0|\partial_k\tilde{H}|1_0\rangle\,\langle 1_0|\tilde{V}|2_0\rangle}{E_{21}} + \frac{\langle 3_0|\partial_k\tilde{H}|0_0\rangle\,\langle 0_0|\tilde{V}|2_0\rangle}{E_{20}}, \tag{S.52}$$

$$\mathrm{Im}\{h_{20}\} = \langle 2_0|\partial_k\tilde{H}|0_0\rangle + \mathcal{O}(1/E^2), \tag{S.53}$$

where we see $\mathrm{Im}\{h_{20}\}$ dominate over $\mathrm{Im}\{h_{32}\}$ since it is not suppressed by energy. In addition, find $\mathrm{Im}\{h_{32}\}$ is maximized near minimum gap point

$$\delta_{2,0} = \pm\sqrt{\delta_1^2 + \Delta_1^2 - \Delta_2^2}. \tag{S.54}$$

The negative pole is suppressed by other factors, such as direct transition, and it is only near the positive pole where we see enhancement in the overall shift current response.

Near the point of minimum energy gap at the band edge, the small ε_{32} in the denominator and diverging $\mathrm{Im}\{h_{20}\}$ in the numerator of Eq. 3 combine to generate an enhanced response in virtual transition. Nevertheless, we remark that the peak shift current response is not located precisely at gap-closing; rather, it is in close proximity to this point, due to the vanishing of transition probability amplitude at the gap-closing point.

Supplementary Note 5: Total Shift Current Response in 2RM

The presence of multiple bands allows for additional transition pairs to happen. The total number of transition pairs is the combination of 2 bands out of all the possible bands, and their contributions need to be summed over when

computing the total shift current response. The total shift current response for $\nu = 0.5$ is given in Fig. Supplementary Figure 2:

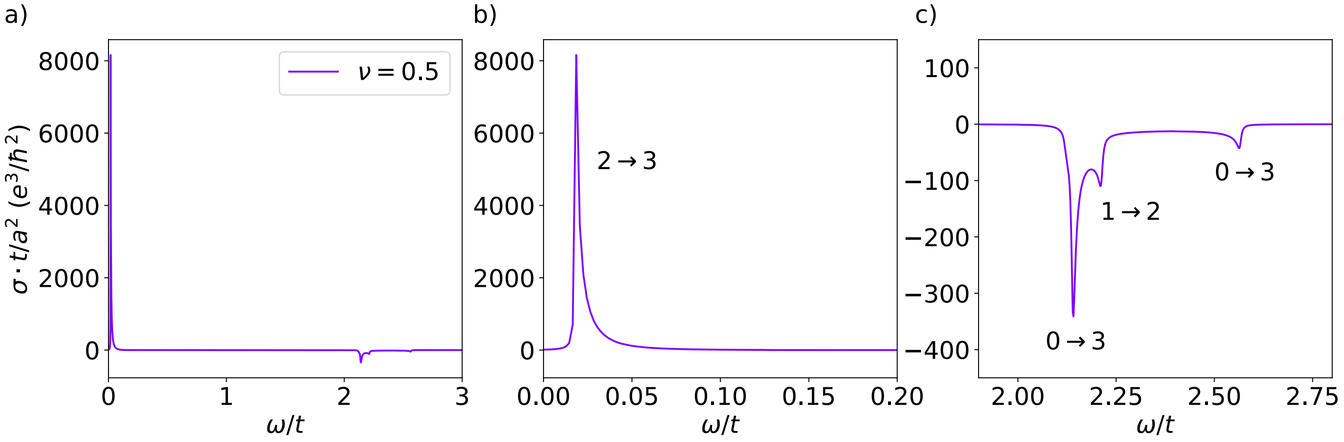

Supplementary Figure 2. Total shift current response for $\nu = 2$ summing up all transition pairs.

The band index is numbered based on Fig. 2a in the main text. Most of the contribution from $\nu = 0.5$ arises from the transition between band 2 and 3, enhanced due to the small band gap. In addition, there are transitions from the first RM model (band 0 to band 3), giving rise to two peaks, corresponding to transition mostly from $ka = \pm\pi$ and $ka = 0$, respectively. The transition from the second RM model (band 1 to band 2) contributes only one peak with contribution mostly from $ka = 0$, which is due to suppression in joint density of state. Summing up all transition pairs also leads to an enhancement in shift current response, compared to the case of simply stacking two pairs of RM chains together (without band mixing).

Supplementary Note 6: Band Mixing in TMG

The multilayer Hamiltonian constructed using ATMG model decouples into pairs of TBG-like bands, but we expect band mixing to occur in actual materials [11, 12]. There are two ways to induce band mixing, either through the application of an external displacement field or through a self-generated displacement field.

External displacement field The effect of the external displacement field is applied through tuning external gate on the device. It modifies the hamiltonian through the addition of the following term in the layer basis:

$$\Delta H = \frac{U}{N-1} \begin{pmatrix} \frac{N-1}{2} & & & & \\ & \frac{N-1}{2} - 1 & & & \\ & & \ddots & & \\ & & & -\frac{N-1}{2} + 1 & \\ & & & & -\frac{N-1}{2} \end{pmatrix} \tag{S.55}$$

where U is the strength of the displacement field, in unit of meV. This leads to band mixing among TBG-like sectors, allowing transition to take place.

Supplementary Note 7: T5G Shift Current Enhancement at Various Fillings

In Fig. 4c in the main text, we showed that both flat-flat and flat-dispersive transitions are enhanced by virtual transitions for $\nu = 2$. The enhancement from virtual transition is present at all fillings, and we include these plots in Fig. Supplementary Figure 3. We note that at charge neutrality ($\nu = 0$), there is an artificial divergence at low frequency due to degeneracy of flat bands at κ points. A finite external displacement field breaks the degeneracy and eliminates such divergence.

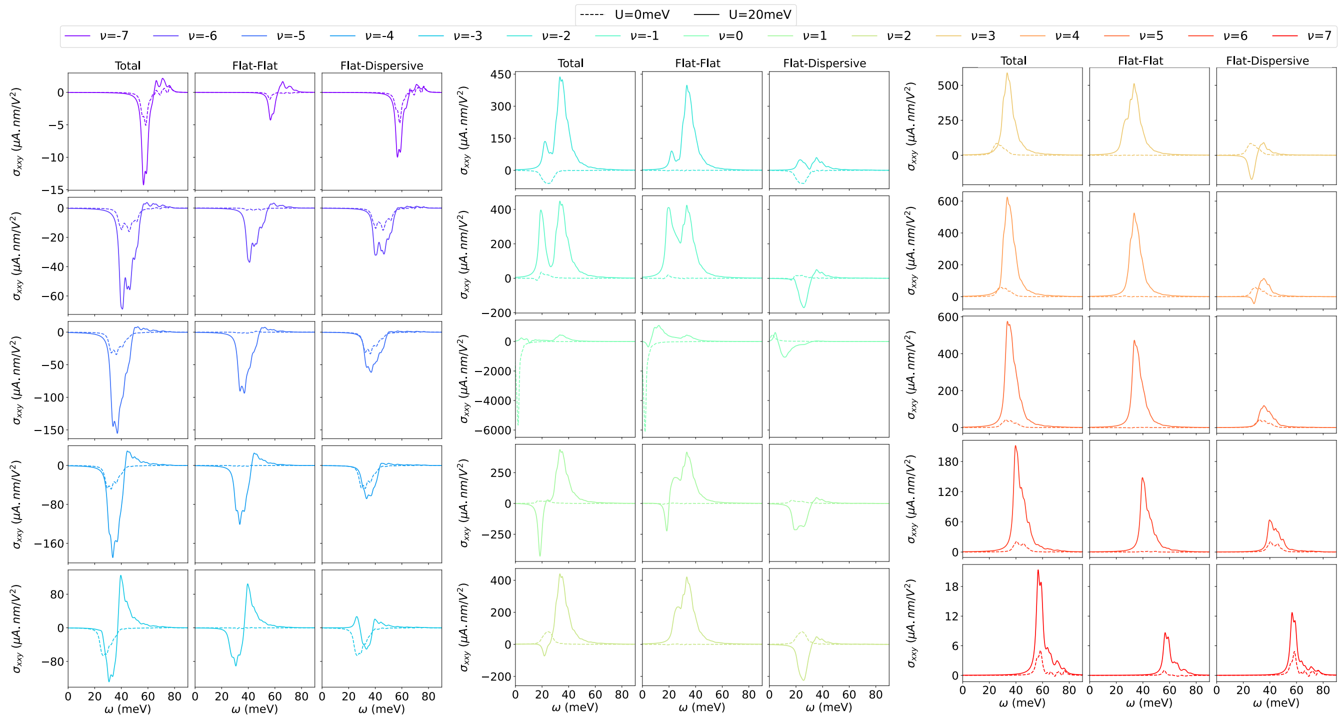

Supplementary Figure 3. T5G at $\theta = 1.32°$ with external displacement $U = 0meV$ (dashed line) and $U = 20meV$ (solid line) for total filling ranging from $\nu = -7$ to $\nu = 7$. We compute the shift current conductivity with transitions between the first flat-band pairs (colored in red in Fig. 4a), separating into flat-flat transitions and flat-dispersive transitions.

Supplementary Note 8: T5G Shift Current and Dependence on Twist Angles

The physical twist angle of the TMG system controls the energy gap of nearby flat bands. At the magic angle where the perfect flat band pair emerges, we expect a large shift current response due to the vanishing of the energy gap. In addition, away from the magic angle in multilayer systems, we anticipate a substantial shift current response stemming from enhanced virtual transition via nearby flat bands. Here we show the dependence of total shift current response $\int dw\sigma_{xxy}(\omega)$ on the physical angle θ_5 of T5G. In addition, we compute the integral of FS metric over k-state as a function of twist angle, as shown in Fig. Supplementary Figure 4. We indeed observe an enhanced shift current response near angles where FS metric diverges, corresponding to a more delocalized Wannier wavefunction.

In the main text, we illustrate virtual transition enhancement in T5G with $\theta_5 = 1.32°$ since the flat bands belong to distinct TBG-like sectors, allowing us to better compare the effect of band mixing on transitions within the same band. As shown in Fig. Supplementary Figure 4b, this angle is not the physical angle that leads to maximized response. The ideal angle occurs when $\theta_5 = 1.28°$, where the two flat bands are tightly intertwined.

At $\theta = 1.28°$, the two pairs of TBG have a similar effective twist angle, but with the top and bottom flat bands flipped with respect to each other. The slight difference in dispersion relations of the top and bottom flat bands results in the intertwining of the bands, as shown in Fig. Supplementary Figure 5. Instead of studying transitions between single band pairs, observing an enhancement in shift current necessitates summing over all transition pairs. Fig. Supplementary Figure 5 b) shows that virtual transitions among the flat bands indeed lead to an enhancement response, and we also observe that $\theta = 1.28°$ generates a larger shift current response than $\theta = 1.32°$.

To search for the optimal twist angles for a higher number of layers, we would want the TBG band pairs to have similar effective angles. A more systematic search can be done by computing the FS metric for the flat bands and pinpointing the angle that gives rise to the largest $\int d^2k \, \mathrm{tr}\, g$. We adopt this procedure and obtained the optimal angles for $N = 6, 7, 8, 9$ layers of twisted graphene, and the result is shown in Fig. Supplementary Figure 6.

The conductivity includes transitions among flat bands and include transition to dispersive bands via virtual transitions. We see that peaks in $\int d^2k \, \mathrm{tr}(g)$ indeed corresponds to the angles where two flat bands are the closest.

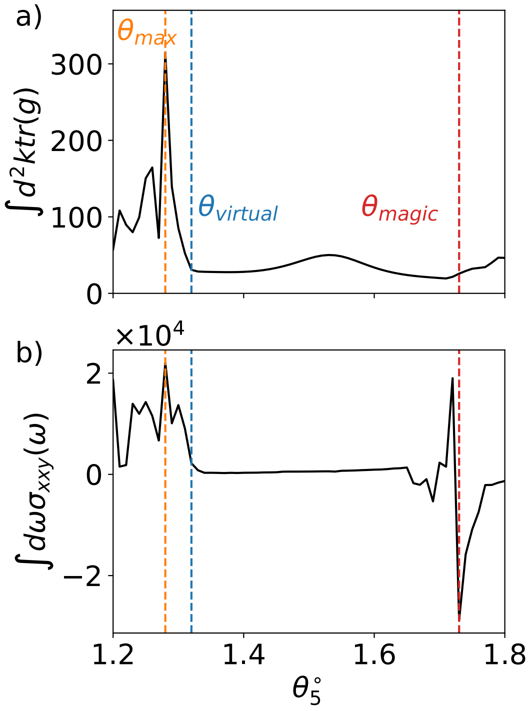

Supplementary Figure 4. T5G FS metric of top flat bands and overall shift current response and their dependence on physical twist angle. The orange dashed line shows the angle that leads to max FS, and correspondingly largest overall shift current measured by metric M. The blue dashed line shows the angle that is used in the main text to illustrate enhancement via virtual transitions. The red line denotes the magic angle, where we also observe an enhancement in the overall shift current response, which we attribute to the increased joint density of states.

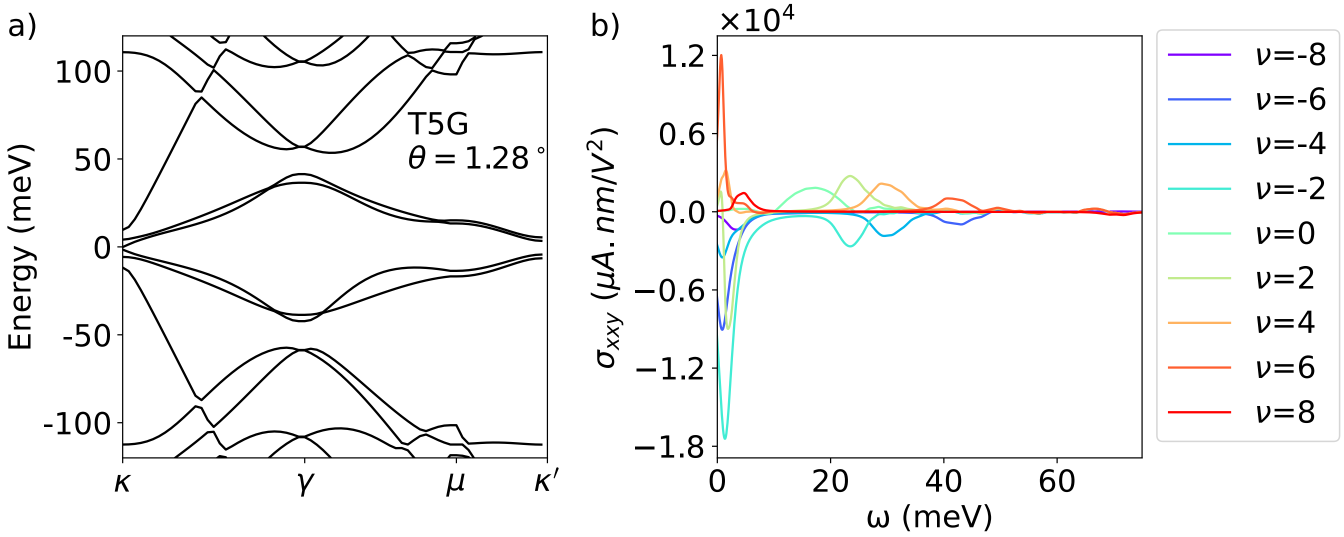

Supplementary Figure 5. T5G band structure occurring at $\theta = 1.28^\circ$ with $U = 20meV$ and the corresponding shift current response at various fillings.

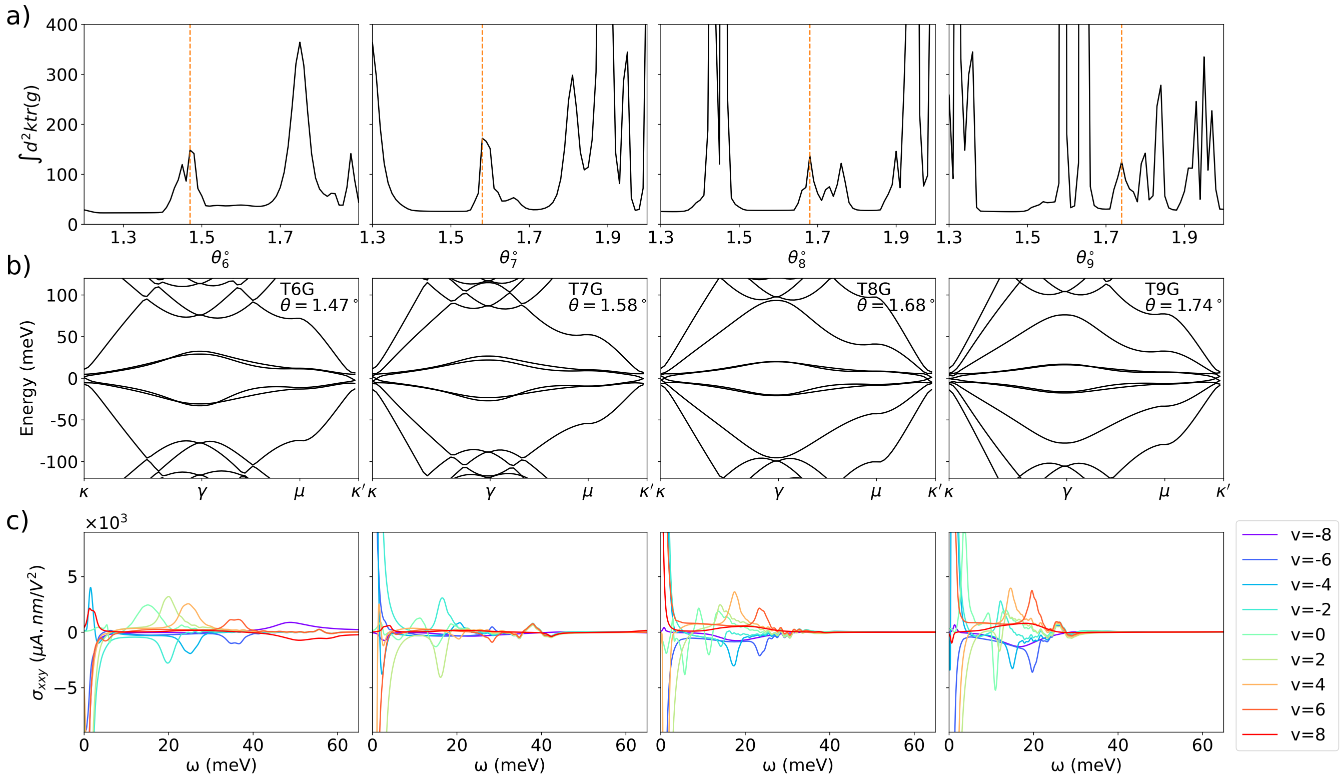

Supplementary Figure 6. Optimal angle for shift current in TMG a) Integral of trace of FS metric for the top flat band of T6G, T7G, T8G, T9G, with the orange dashed line indicating the optimal angle. b) Their corresponding band structure at optimal angles and c) their conductivity $\sigma_{xxy}(\omega)$ for a wide range of fillings.

Supplementary Note 9: T5G Symmetry

The TMG model has C_{3z} symmetry, which restricted $\sigma^{\beta}_{\alpha\alpha}$ to two independent components. A detailed argument is discussed in [13] for TBG, so here we simply restate the result for TMG. We have

$$\sigma^y_{xx} = -\sigma^y_{yy} = \sigma^y_{xy} = \sigma^x_{yx} \neq 0, \tag{S.56}$$

$$\sigma^x_{yy} = -\sigma^x_{xx} = \sigma^y_{yx} = \sigma^y_{xy} \neq 0. \tag{S.57}$$

In the main text, we focused on σ^y_{xx} component.

Supplementary Note 10: Finite Broadening at Low-Frequency

We point out that the non-zero shift current conductivity at low-frequency is due to finite Lorentzian broadening. In computing shift current conductivity, we need to evaluate $\delta(\omega - \varepsilon_{mn})$ in Eq. 2. We choose to approximate the delta function using

$$\delta(x) \approx \frac{\gamma}{\pi(x^2 + \gamma^2)}, \tag{S.58}$$

where γ characterizes the width of the function. We choose γ to be the average energy spacing of the participating bands between nearby k-points in the Brillouin zone. This large choice of γ ensures lack of spurious (due to a finite mesh size) oscillations in the conductivity from Fig. 1e at finite frequencies, however it also renders our conductivity finate as $\omega \to 0$ where the contributions stem from the two closely spaced flat bands. With higher mesh resolution, conductivity as $\omega \to 0$ vanishes as required.

Supplementary References

[1] D. E. Parker, T. Morimoto, J. Orenstein, and J. E. Moore, Diagrammatic approach to nonlinear optical response with application to weyl semimetals, Phys. Rev. B **99**, 045121 (2019).

[2] A. M. Cook, B. M. Fregoso, F. De Juan, S. Coh, and J. E. Moore, Design principles for shift current photovoltaics, Nature communications **8**, 1 (2017).

[3] J. E. Sipe and A. I. Shkrebtii, Second-order optical response in semiconductors, Phys. Rev. B **61**, 5337 (2000).

[4] J. P. Provost and G. Vallee, Riemannian structure on manifolds of quantum states, Communications in Mathematical Physics **76**, 289–301 (1980).

[5] P. Törmä, Essay: Where can quantum geometry lead us?, Phys. Rev. Lett. **131**, 240001 (2023).

[6] F. Xie, Z. Song, B. Lian, and B. A. Bernevig, Topology-bounded superfluid weight in twisted bilayer graphene, Phys. Rev. Lett. **124**, 167002 (2020).

[7] M. Iskin, Effective-mass tensor of the two-body bound states and the quantum-metric tensor of the underlying bloch states in multiband lattices, Phys. Rev. A **105**, 023312 (2022).

[8] Z. Dai and A. M. Rappe, Recent progress in the theory of bulk photovoltaic effect, Chemical Physics Reviews **4**, 10.1063/5.0101513 (2023).

[9] B. M. Fregoso, T. Morimoto, and J. E. Moore, Quantitative relationship between polarization differences and the zone-averaged shift photocurrent, Phys. Rev. B **96**, 075421 (2017).

[10] W. P. Su, J. R. Schrieffer, and A. J. Heeger, Solitons in polyacetylene, Phys. Rev. Lett. **42**, 1698 (1979).

[11] Y. Zhang, R. Polski, C. Lewandowski, A. Thomson, Y. Peng, Y. Choi, H. Kim, K. Watanabe, T. Taniguchi, J. Alicea, *et al.*, Ascendance of superconductivity in magic-angle graphene multilayers, arXiv:2112.09270 (2021).

[12] K. c. v. Kolář, Y. Zhang, S. Nadj-Perge, F. von Oppen, and C. Lewandowski, Electrostatic fate of n-layer moiré graphene, Phys. Rev. B **108**, 195148 (2023).

[13] S. Chaudhary, C. Lewandowski, and G. Refael, Shift-current response as a probe of quantum geometry and electron-electron interactions in twisted bilayer graphene, Phys. Rev. Res. **4**, 013164 (2022).
